# Direct Exchange of Oxygen and Selenium Atoms in the 1,2,5-Oxadiazoles and 1,2,5-Selenadiazoles by Action of Sulfur Monochloride

**DOI:** 10.3390/molecules200814522

**Published:** 2015-08-12

**Authors:** Lidia S. Konstantinova, Ekaterina A. Knyazeva, Oleg A. Rakitin

**Affiliations:** 1 N. D. Zelinsky Institute of Organic Chemistry, Russian Academy of Sciences, 119991 Moscow, Russia; E-Mails: konstantinova_ls@mail.ru (L.S.K.); katerina_knyazev@mail.ru (E.A.K.); 2 Department of Chemistry, South Ural State University, 454080 Chelyabinsk, Russia

**Keywords:** 1,2,5-thiadiazoles, 1,2,5-oxadiazoles, 1,2,5-selenadiazoles, sulfur monochloride

## Abstract

A short synthetic approach to fused 1,2,5-thiadiazoles from the corresponding 1,2,5-oxadiazoles and 1,2,5-selenadiazoles has been developed. Mono- and bis(1,2,5-thiadiazoles) were selectively obtained in high yields. The pathways for these novel reactions were discussed.

## 1. Introduction

1,2,5-Thiadiazoles and particularly their benzo-fused derivatives have been known for many years, and their synthesis and applications in various branches of technology and medicine were extensively investigated and reviewed [1,2,3,4,5]. Recently, they were found to be an efficient electron acceptor and were used as the building blocks of many actual or potential molecule-based functional materials for organic electronics and spintronics [6,7,8,9,10,11,12]. Although methods for the preparation of fused 1,2,5-thiadiazoles are numerous and well elaborated [2,3,5], there is still a lack of suitable preparative approaches to many interesting derivatives containing electron-deficient heterocycles or electron-withdrawing groups.

**Scheme 1:**
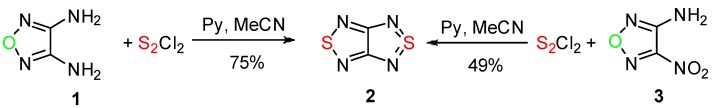
Synthesis of [1,2,5]thiadiazolo[3,4-*c*][1,2,5]thiadiazole **2**.

A few years ago 3,4-diamino-1,2,5-oxadiazole **1** was found to react with sulfur monochloride and pyridine in acetonitrile and gave, unexpectedly, [1,2,5]thiadiazolo[3,4-*c*][1,2,5]thiadiazole **2** in high yield (Scheme 1) [10]. The main feature of this transformation is an exchange of the oxygen atom in the 1,2,5-oxadiazole ring with a sulfur atom in the reaction with sulfur monochloride. To the best of our knowledge, this is the first example of this unusual reaction. Later on, it was shown that another oxadiazole derivative, 4-amino-3-nitro-1,2,5-oxadiazole **3**, can undergo a similar transformation with S_2_Cl_2_ in lower yield [13].

**Scheme 2:**
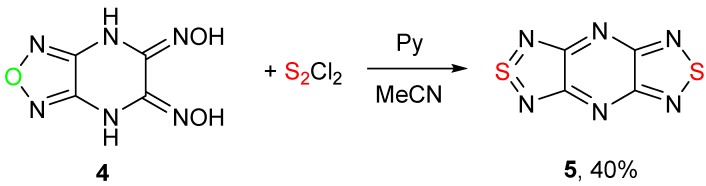
Synthesis of bis([1,2,5]thiadiazolo)-[3,4-*b*;3′,4′-*e*]pyrazine **5**.

Recently, it was discovered that under similar conditions, (5*Z*,6*Z*)-[1,2,5]oxadiazolo[3,4-*b*]pyrazine-5,6(4*H*,7*H*)-dione dioxime **4** gave tricyclic bis([1,2,5]thiadiazolo)-[3,4-*b*;3′,4′-*e*]pyrazine **5** in moderate yield (Scheme 2). In that case, three reactions occurred: exchange of an oxygen atom in a 1,2,5-oxadiazole ring by a sulfur atom, formation of thiadiazole ring from dioxime, and aromatization of a piperazine ring [14].

A specific feature of sulfur monochloride (S_2_Cl_2_) is its diverse reactivity [15,16,17,18]. The most useful property of this compound is its sulfurating ability. Apart from this, S_2_Cl_2_ is a strong chlorinating agent, but it is rarely used since there are many other chlorinating agents that afford higher yields of the reaction products. The oxidative ability of S_2_Cl_2_ has been investigated to a much lower extent because the products of formal oxidation reactions are formed, as a rule, in a course of complex multistage processes involving chlorination, dehydrochlorination, sulfuration, *etc.* A direct exchange of a chalcogen atom with a sulfur atom in the reaction with sulfur monochloride in chalcogen-nitrogen heterocycles has not been discovered before our works [10,13]. To the best of our knowledge, the only formal analogy of this exchange is the classical Yuryev reaction performed under very drastic conditions [19,20,21]. However, the reaction pathways seem to be very different and its scope is very limited to heterocycles with one heteroatom, and high yields were achieved by using furan as a starting material; vigorous conditions of this method did not allow its spread for compounds with labile groups.

In this paper, we report a study of a reaction between a wide range of 1,2,5-oxadiazoles and 1,2,5-selenadiazoles and sulfur monochloride for the synthesis of fused mono- and bis(1,2,5-thiadiazoles).

## 2. Results and Discussion

### 2.1. Conversion of 1,2,5-Oxadiazoles into 1,2,5-Thiadiazoles

**Scheme 3:**
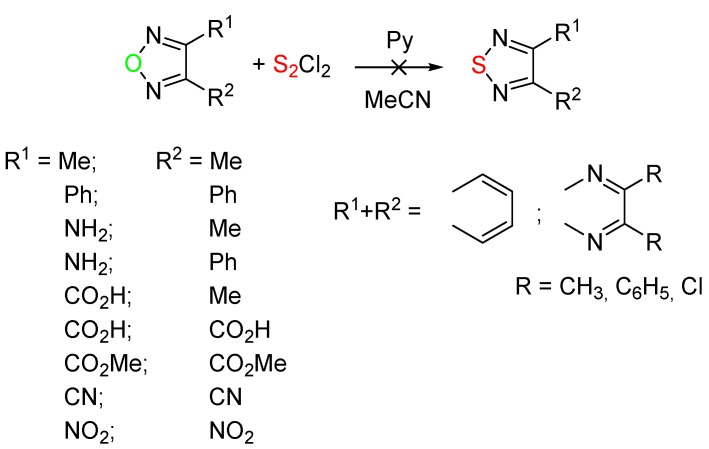
1,2,5-Oxadiazoles which did not react with sulfur monochloride.

We attempted to extend the reaction with sulfur monochloride and pyridine in acetonitrile to other monocyclic, benzo-, and pyrido-fused 1,2,5-oxadiazoles. It was found that the uncondensed 1,2,5-oxadiazoles, 2,1,3-benzooxadiazole, and 5,6-disubstituted [1,2,5]oxadiazolo[3,4-*b*]pyrazines, shown in Scheme 3, did not react with S_2_Cl_2_ under these conditions in practically all cases. Employing 1,4-diazabicyclo[2.2.2]octane or triethylamine as a base and changing the solvent to chloroform or to dimethylformamide at temperatures from −25 to 100 °C also did not lead to 1,2,5-thiadiazole derivatives. The analysis of these results has driven us to the conclusion that for the successful conversion of 1,2,5-oxadiazoles into 1,2,5-thiadiazoles, one or two NH_2_ or NH groups attached to oxadiazole ring are needed.

**Scheme 4:**
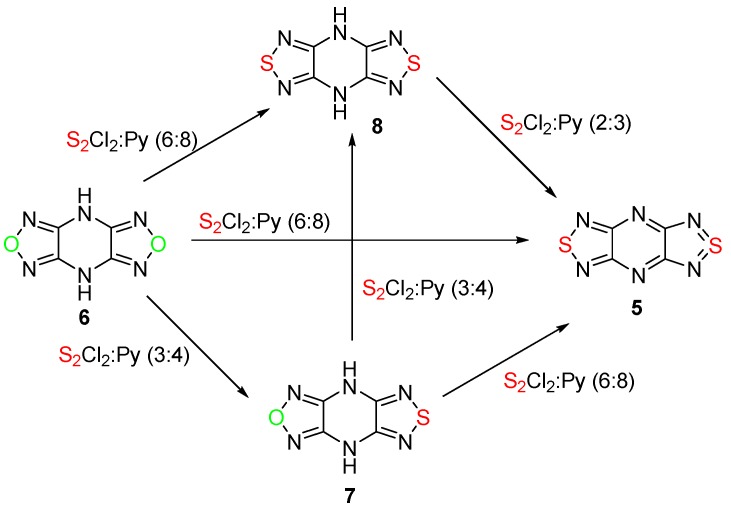
Reactions of 4*H*,8*H*-bis[1,2,5]oxadiazolo[3,4-*b*:3′,4′-*e*]pyrazine **6** with sulfur monochloride in MeCN.

To continue our attempts to convert the 1,2,5-oxadiazole ring into 1,2,5-thiadiazole, the reaction of 4*H*,8*H*-bis[1,2,5]oxadiazolo[3,4-*b*:3′,4′-*e*]pyrazine **6**, containing two oxadiazole rings and two NH groups, with sulfur monochloride was investigated in detail (Scheme 4).

It was found that the treatment of compound **6** with an excess of sulfur monochloride (6 equiv) and pyridine (8 equiv) in boiling MeCN gave aromatic bis(1,2,5-thiadiazole) **5** in good yield (Scheme 4, Table 1, entry 1).

**Table 1:** Reactions of [1,2,5]oxadiazolo[3,4-*b*]pyrazines **6**–**8** with S_2_Cl_2_ in MeCN.

No.	Starting Compound	Quantity of Reagents for 1 Equiv of Starting Compound		Temperature, °С	Reaction Time, h	Reaction Product, Yield %
		S_2_Cl_2_	Pyridine			
1	**6**	6	8	82	4	**5**, 76
2	**6**	3	4	20	20	**7**, 90
3	**6**	6	8	20	20	**8**, 83
4	**7**	3	4	20	20	**8**, 66
5	**7**	6	8	82	5	**5**, 56
6	**8**	2	3	82	2	**5**, 85

The formation of thiadiazoles **7** and **8** from bis(1,2,5-oxadiazole) **6** indicated that the successive formation of two thiadiazole rings occurred at room temperature (entries 2 and 3, Table 1) and preceded the final aromatization of the piperazine cycle in boiling MeCN (Scheme 4). This was confirmed by the treatment of oxadiazolothiadiazole **7** with the same mixture at room temperature which led to bis(1,2,5-thiadiazole) derivative **8** in good yield (entry 4, Table 1), and the conversion of bis(1,2,5-thiadiazole) **8** to aromatic tricycle **5** at high temperature (entry 6, Table 1).

**Scheme 5:**
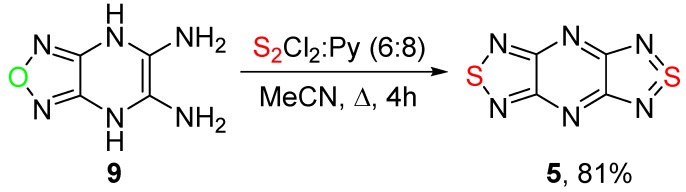
Reaction of 4,7-dihydro[1,2,5]oxadiazolo[3,4-*b*]pyrazine-5,6-diamine **9** with S_2_Cl_2_.

Tricycle **5** can be obtained also from diamine derivative **9** by treatment with S_2_Cl_2_ and pyridine in boiling MeCN (Scheme 5). In that case, two processes take place simultaneously—the formation of the thiadiazole ring from the diamine moiety and the dehydration of dihydropiperazine into the aromatic pyrazine ring, presumably by action of sulfur monochloride.

Dehydration of dihydropiperazine to a pyrazine ring is quite a rare process and it was investigated in detail for bis(1,2,5-thiadiazole) derivative **8**. It was found that the treatment with a rank of oxidizers usually used for similar processes—bromine, DDQ, PhI(OAc)_2_ and its perfluoro analog PhI(O(C(O)CF_3_))_2_ in various solvents (CHCl_3_, THF, ether, MeCN)—gave no reaction in most cases, and in some it led to the decomposition of starting material. Aromatic bis-1,2,5-thidiazolopyrazine **5** has been obtained in the reaction of **8** with sulfuryl chloride, and it was found that to get a high yield of compound **5**, it is necessary to employ eight equivalents of oxidant and hours-long boiling.

**Scheme 6:**
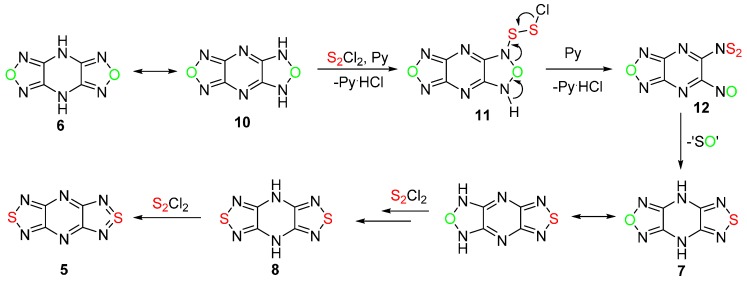
A plausible mechanism for the formation of bis([1,2,5]thiadiazolo)-[3,4-*b*;3′,4′-*e*]pyrazine **5** from 4*H*,8*H*-bis[1,2,5]oxadiazolo[3,4-*b*:3′,4′-*e*]pyrazine **6**.

The described procedures provide a new synthetic pathway to fused 1,2,5-thiadiazoles from corresponding oxadiazoles. The key steps may be explained by the sulfurization of the tautomeric form **10** of the tricycle **6** with sulfur monochloride in the presence of the base to give chlorodithio derivative **11**, followed by the elimination of hydrogen chloride with the formation of *N*-thiosulfinylamine **12** (Scheme 6). Further cyclization of compound **12** into 1,2,5-thiadiazole **7** via cycloaddition/retrocycloaddition with the extrusion of sulfur monoxide (SO), which is thermodynamically unstable and decomposes very rapidly [22], may occur. A similar formation of the 1,2,5-thiadiazole ring from *N*-thiosulfinylamine and nitro groups has recently been proposed [13]. This reaction could then be repeated to give bis(1,2,5-thiadiazole) **8**, which may oxidize with the formation of aromatic tricycle **5**. Bis([1,2,5]thiadiazolo)-[3,4-*b*;3′,4′-*e*]pyrazine **5** is of interest as a prominent precursor of persistent radical anions [6].

### 2.2. Conversion of 1,2,5-Selenadiazoles into 1,2,5-Thiadiazoles

**Scheme 7:**
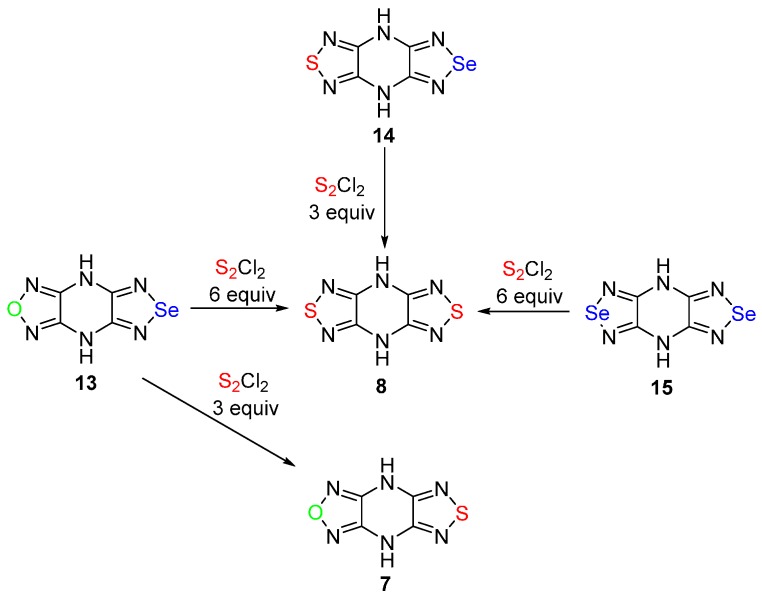
Reactions of [1,2,5]selenadiazolo[3,4-*b*]pyrazines **13**–**15** with sulfur monochloride.

To further explore the possibility of sulfur monochloride to substitute the oxygen atom in 1,2,5-oxadiazoles with a sulfur atom, the reaction of 4,8-dihydro[1,2,5]oxadiazolo[3,4-*b*][1,2,5]selenadiazolo[3,4-*e*]pyrazine **13** with S_2_Cl_2_ was investigated. Tricycle **13** was found to be inactive by treatment with S_2_Cl_2_ in MeCN, apparently because it was not dissolved in the reaction mixture. When MeCN was substituted with DMF, 4,8-dihydro[1,2,5]oxadiazolo[3,4-*b*][1,2,5]thiadiazolo[3,4-*e*]pyrazine **7** was isolated in high yield (entry 1, Table 2). It means that a selective exchange of the selenium atom in the 1,2,5-selenadiazole ring with a sulfur atom has occurred. There is only one example of conversion of 1,2,5-selenadiazoles to 1,2,5-thiadiazoles by the action of hydrogen sulfide to the (1,2,5-selenadiazolo)porphyrazine derivative [23]. When an excess of sulfur monochloride was used in the reaction of pyrazine **13**, both selenium and oxygen atoms were replaced by sulfur atoms with the formation of bis(1,2,5-thiadiazole) **8** (entry 2, Table 2). Also, mono- and bis(1,2,5-selenadiazoles) **14** and **15**, respectively, can be involved in this reaction to give bis(1,2,5-thiadiazole) **8** in high yields (Scheme 7).

The most important results are summarized in Table 2.

**Scheme 8:**
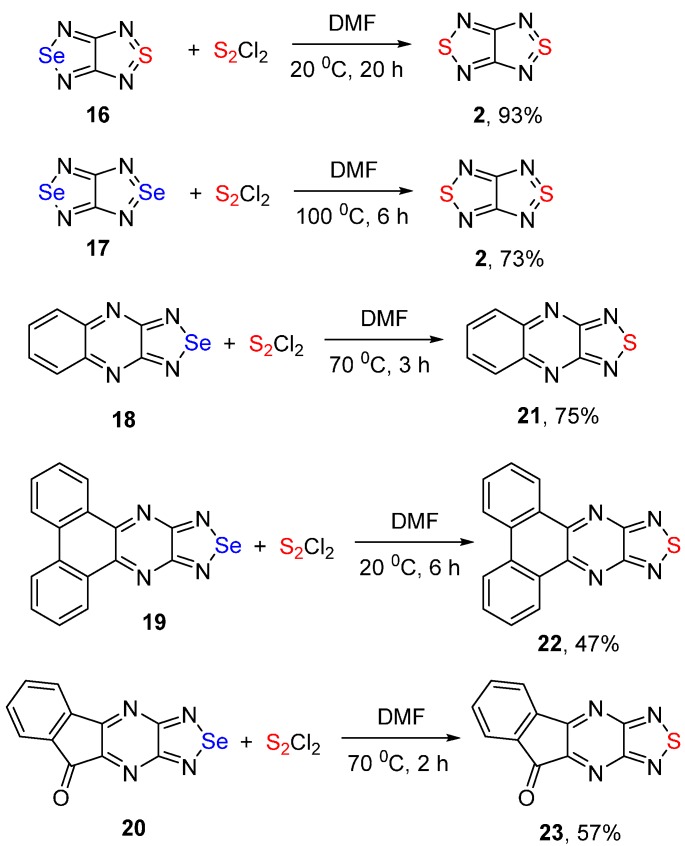
Reactions of fused 1,2,5-selenadiazoles **16**–**20** with sulfur monochloride.

In an attempt to develop general direct synthesis of 1,2,5-thiadiazoles from selenadiazoles, the latter were reacted with S_2_Cl_2_. Treatment of 1,2,5-selenadiazoles fused with electron-acceptor heterocycles, such as 1,2,5-thiadiazole (**16**), 1,2,5-selenadiazole (**17**), quinoxaline (**18**), and others (**19**–**20**) with S_2_Cl_2_ in DMF, gave the corresponding mono- and bis(1,2,5-thiadiazoles) (**2**, **21**–**23**) at various temperatures in good yields (Scheme 8, entries 5–9, Table 2).

**Table 2:** Reactions of fused 1,2,5-selenadiazoles with S_2_Cl_2_ in DMF.

No.	Starting Compound	Quantity of S_2_Cl_2_ for 1 Equiv of Starting Compound	Тemperature, °С	Reaction Time, h	Reaction Product, Yield %
1	**13**	3	20	3	**7**, 91
2	**13**	6	100	6	**8**, 78
3	**14**	3	20	20	**8**, 83
4	**15**	6	100	6	**8**, 85
5	**16**	3	20	20	**2**, 93
6	**17**	6	100	6	**2**, 73
7	**18**	2	70	3	**21**, 75
8	**19**	2	20	6	**22**, 47
9	**20**	2	70	2	**23**, 57

In all cases, the formation of the characteristic red amorphous precipitate of elemental selenium was indicated. In the case of **16**, this precipitate was isolated in practically quantitative yield and its structure was confirmed by mass-spectrometry and elemental analysis.

We attempted to extend this reaction to benzo-fused and monocyclic 1,2,5-selenadiazoles. It was found that 2,1,3-benzoselenadiazole and 3,4-diphenyl-1,2,5-selenadiazole did not react with sulfur monochloride in organic solvents under forcing conditions (refluxing in acetonitrile or heating in DMF at 100 °C for 10 h) and were recovered from the reaction mixtures in practically quantitative yields.

**Scheme 9:**
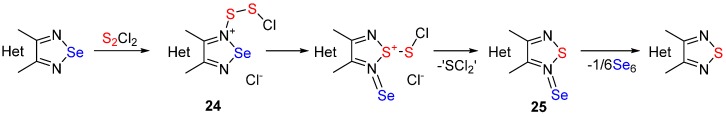
A plausible mechanism for the transformation of 1,2,5-selenadiazoles into 1,2,5-thiadiazoles.

To compare the reactivity of 1,2,5-oxadiazoles and 1,2,5-selenadiazoles in the reaction with sulfur monochloride, it should be noted that in both cases a fused nitrogen-containing heterocycle is needed for the successful exchange of oxygen or selenium atoms to sulfur. The most plausible mechanism of the conversion of 1,2,5-selenadiazoles into 1,2,5-thiadiazoles is shown in Scheme 9. The key steps may include the sulfurization of the selenadiazole ring with sulfur monochloride to give chlorodithio derivative **24**, followed by the recyclization of the selenadiazole ring and the elimination of sulfur dichloride with the formation of 1,2,5-selenadiazolo-*N*-selenide **25** (Scheme 9). Further extrusion of elemental selenium, which is precipitated from the reaction mixture, affords the final 1,2,5-thiadiazole.

The method described here supplemented and enhanced the possibilities for the synthesis of fused 1,2,5-thiadiazoles.

## 3. Experimental Section

### 3.1. General Information

Elemental analyses for C, H, and N were performed with Perkin Elmer 2400 Elemental Analyser (Perkin Elmer, Waltham, MA, USA). Melting points were determined on a Boetius hot-stage apparatus and are uncorrected.

^1^H- (300.1 MHz) and ^13^C- (75.5 MHz) NMR spectra were taken for CDCl_3_ solutions (unless otherwise indicated) with a Bruker AM-300 (Bruker AXS Handheld Inc., Kennewick, WA, USA) and referred to tetramethylsilane (^1^H and ^13^C), *J* values are given in Hz.

MS spectra (EI, 70 eV) were obtained with a Finnigan MAT INCOS 50 (Hazlet, NJ, USA). High-resolution MS spectra were measured on a Bruker micrOTOF II instrument (Bruker Daltonik Gmbh, Bremen, Germany) using electrospray ionization (ESI). The measurement was operated in a positive ion mode (interface capillary voltage −4500 V) or in a negative ion mode (3200 V); mass range was from *m*/*z* 50 to *m*/*z* 3000 Da; external or internal calibration was done with Electrospray Calibrant Solution (Fluka). A syringe injection was used for solutions in acetonitrile, methanol, or water (flow rate 3 µL·min^−1^). Nitrogen was applied as a dry gas; interface temperature was set at 180 °C.

IR spectra were measured with a Specord M-80 (Carl Zeiss, Jena, Germany) instrument in KBr pellets.

Fused 1,2,5-oxadiazoles **6** [24], **7** [25], **9** [25] and 1,2,5-selenadiazoles **13**–**20** [11,26,27] were prepared according to the published procedures.

### 3.2. General Procedure for the Reaction of 1,2,5-Oxadiazoles with S_2_Cl_2_ and Pyridine in Acetonitrile

Sulfur monochloride (quantity, see Table 1) was added dropwise to a stirred suspension of oxadiazole **6**–**9** (1.0 mmol) and pyridine (quantity, see Table 1) in dry acetonitrile (10 mL) under argon at −25 °C. The mixture was stirred at room temperature or heated at the temperature and for the time specified in Table 1. The precipitate was filtered and washed thoroughly with MeCN and hexane.

*Bis([1,2,5]Thiadiazolo)-[3,4-b;3',4'-e]pyrazine* **5**. Yellow solid, mp 320–323 °С (Lit. [14] mp 320–322 °С). IR and mass spectra are similar to the literature data [14].

*4,8-Dihydrobis([1,2,5]thiadiazolo)[3,4-b:3',4'-e]pyrazine* **8**. Beige crystals, mp >350 °С. Anal. calcd for С_4_Н_2_N_6_S_2_. (198.23): C, 24.24; H, 1.02; N, 42.40; S, 32.35. Found: C, 24.38; H, 0.98; N, 42.26; S, 32.38. NMR (DMSO-*d*_6_), δ, ^1^H: 10.11 (s, 2Н, NH); ^13^С: 142.8, 155.1. IR, ν, cm^−1^: 3136 (NH), 1628, 1334, 941, 520. MS, *m*/*z* (%): 198 (M^+^, 100), 171 (14), 131 (7), 46 (9). ESI-MS: found *m*/*z* 220.9850; calc. for С_4_Н_2_N_6_S_2_ [M + Na]^+^ 220.9675.

### 3.3. General Procedure for the Reaction of 1,2,5-Selenadiazoles with S_2_Cl_2_ in DMF

Sulfur monochloride (quantity, see Table 2) was added dropwise to a stirred suspension of selenadiazole **13**–**20** (1.0 mmol) in dry DMF (10 mL) under argon at −5 °C. The mixture was stirred at room temperature or heated at the temperature and for the time specified in Table 2. Elemental selenium was filtered, the reaction mixture was poured into H_2_O with ice, extracted with EtOAc (4 mL × 20 mL). Combined extracts were washed with brine, dried over MgSO_4,_ and the solvent was evaporated under reduced pressure. Yields are given in Table 2.

*[1,2,5]Thiadiazolo[3,4-b]quinoxaline* **21**. Yellow solid, mp 195–197 °С (Lit. [28] mp 199.5–200.5 °С). IR and mass spectra are similar to the literature data [28].

*Dibenzo[f,h][1,2,5]thiadiazolo[3,4-b]quinoxaline* **22**. Yellow solid, mp 302–304 °С (Lit. [28] mp 298–300 °С). IR and mass spectra are similar to the literature data [28].

*9Н-Inden[1,2-e][1,2,5]selenadiazolo[3,4-b]pyrazin-9-one* **23**. Yellow solid, mp 286–287 °С. Anal. calcd for С_11_Н_4_N_4_OS (240.24): С 54.99, Н 1.68, N 23.32. Found: C 55.13, H 1.82, N 23.09. NMR (DMSO-*d*_6_), δ, ^1^H: 7.84 (m, 1Н, Ph), 7.97 (m, 2Н, Ph), 8.20 (d, 1Н, *J* = 7.3 Hz, Ph); ^13^С: 126.6, 127.7, 137.6, 140.8 (4 CH), 133.3, 138.0, 141.7, 143.3, 158.7, 163.9 (6 sp^2^ tertiary C), 190.3 (C=O). ESI-MS: found *m*/*z* 241.0177; calc. for С_11_Н_4_N_4_OS [M]^+^ 241.0179.

## 4. Conclusions

A new reaction, namely that of fused 1,2,5-oxadiazoles and 1,2,5-selenadiazoles with sulfur monochloride, has been described as a one-pot synthetic route to 1,2,5-thiadiazoles. This procedure is especially valuable in cases where 1,2,5-oxadiazoles (e.g., **6**) are more readily available than the corresponding 1,2,5-thiadiazoles. These reactions have broadened the utility of sulfur monochloride as a highly efficient sulfurating reagent. Fused 1,2,5-thiadiazoles are of interest as precursors of persistent radical anions and components of charge-transfer complexes.
